# Herbal formula YYJD inhibits tumor growth by inducing cell cycle arrest and senescence in lung cancer

**DOI:** 10.1038/s41598-017-05146-x

**Published:** 2017-07-10

**Authors:** Tingting Zheng, Zujun Que, Lijing Jiao, Yani Kang, Yabin Gong, Jialin Yao, Chao Ma, Ling Bi, Qihan Dong, Xiaodong Zhao, Ling Xu

**Affiliations:** 1 Cancer Institute of Traditional Chinese Medicine, Shanghai University of Traditional Chinese Medicine, 725 South Wanping Rd, Shanghai, 200032 China; 2 Department of Oncology, Yueyang Hospital of Integrated Traditional Chinese and Western Medicine, Shanghai University of Traditional Chinese Medicine, 110 Ganhe Rd, Shanghai, 200437 China; 3 Institute of Clinical Immunology, YueYang Hosptial of Intergrated Traditional Chinese and Western Medicine, Shanghai University of Traditional Chinese Medicine, 110 Ganhe Rd, Shanghai, 200437 China; 4 School of Biomedical Engineering and Bio-ID Center, Shanghai Jiao To ng University, 800 Dongchuan Rd, Shanghai, 200240 China; 5 Endocrinology Section, Central Clinical School and Charles Perking Center, The University of Sydney, School of Science and Health, The Western Sydney University, Sydney, Australia

## Abstract

Lung cancer represents a major cause of cancer-related death worldwide. Although various tactics and anti-tumor drugs have been used to improve curative effects, five-year survival rate of lung cancer patients remains poor. In this study, we investigated the action and underlying mechanisms of our recently optimized Chinese herbal formula Yangyinjiedu (YYJD) against lung cancer. YYJD significantly inhibits the proliferation of lung cancer cell lines (95-D, A549, H460 and H1975) by inducing cell cycle arrest and senescence in a dose-dependent manner. In particular, YYJD induces significant G2/M phase arrest and inhibits the colony formation of lung cancer cells. Moreover, we found that administration of YYJD could inhibit the growth of xenografted lung cancer cells in nude mice without loss in body weight. Our findings suggest that the herbal formula YYJD is a potential anti-tumor agent against lung cancer.

## Introduction

Lung cancer is one of the most commonly diagnosed malignant tumors and remains the major cause of cancer-related death in the world^1^. In China, lung cancer accounts for about one-sixth of all new cancer cases, and the death rate is 6.102/1000, accounting for over one-fifth of all tumor deaths^2^. Even though new treatment approaches are emerging, the 5-year survival rate is less than 20%^3^. The poor prognosis highlights the importance to develop novel drugs with high efficiency for the treatment of lung cancer^4^.

Chinese Herb Medicine has been used to treat human diseases for hundreds of years. In modern era, combined with chemotherapy, it plays an important role in the treatment of cancer, including enhancing the effect of chemotherapy, patient pain relief and prolonging patient survival time^5–7^. Jinfukang is a herb formula and has been clinically used to treat human lung cancer patients for many years. Previous studies have revealed that Jinfukang inhibits tumor growth in mice model of lung cancer, suppresses cellular proliferation and induces apoptosis of lung cancer cells^8–10^. However, the twelve herbs in the formula make it difficult to elucidate the anti-tumor mechanisms. Recently, some computational algorithms have been developed to optimize the number of ingredients in the formula without compromising its efficacy^11^. Adopting a similar strategy, we are able to decrease from original twelve herbs of Jinfukang to five, consisting of Astragalus, Radix Ophiopogonis, Paris polyphylla, Glossy Privet Fruit and Fiveleaf Gynostemma^12^. The optimized resulting formula is named as Yangyinjiedu (YYJD). In this study, we examined the mechanism of anti-tumor effect of YYJD and found that YYJD exerted anti-proliferation and pro-senescence effects in lung cancer cells. In addition, it suppressed growth of lung cancer-xenograft in nude mice.

## Results

### YYJD inhibits proliferation of lung cancer cells

**Figure 1:**
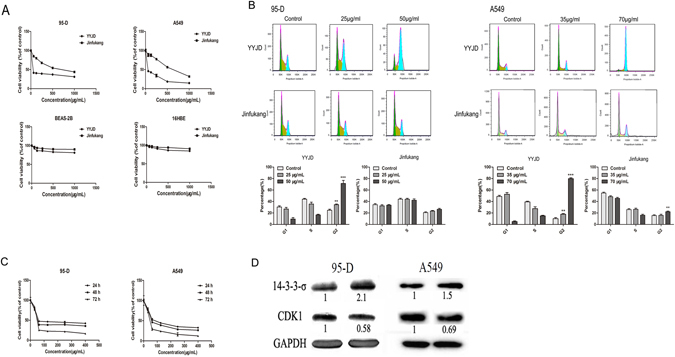
YYJD inhibits the proliferation and induces cell cycle arrest at G2/M phase of lung cancer cells. (**A**) The effects of YYJD and Jinfukang on 95-D, A549, BEAS-2B and 16HBE cells proliferation after 24 h treatment. (**B**) YYJD and Jinfukang induce cell cycle arrest at G2/M phase, and the effects of YYJD and Jinfukang on 95-D and A549 cells were analyzed by flow cytometry. (**C**) The effects of various concentrations of YYJD on 95-D and A549 cells viability after 24 h, 48 h and 72 h treatment. (**D**) The expression levels of cell cycle relevant proteins. The results are expressed with the mean ± SD for at least three independent experiments. *P < 0.05, **P < 0.01 and ***P < 0.001 compared with control group (culture medium only).

YYJD is a new formula optimized from our previously published Jinfukang^8–10, 13^ using Placket-Burman and random forests algorithm^12^. The half maximal inhibitory concentration (IC50) of YYJD and Jinfukang were 70 µg/ml and 570 µg/ml respectively in A549 cell, and 50 µg/ml and 640 µg/ml respectively in 95-D cell, determined by CCK8 cell viability assay. The impact of YYJD and Jinfukang in normal lung cells (BEAS-2B cell and 16HBE) was much smaller (Fig. 1A).

Cell cycle analysis revealed that the proportion of lung cancer cells in G2/M phase was significantly increased in a dose-dependent manner upon YYJD treatment for 24 h, while the proportion of lung cancer cells in G1 and S phases was decreased (Fig. 1B). Although there were some indications of an increase in G2/M following treatment with Jinfukang, its effect was smaller than YYJD.

To verify the effect of YYJD on lung cancer cell proliferation, 95-D and A549 were treated with YYJD at different concentrations for 24 h, 48 h and 72 h, respectively. The proliferation of lung cells was significantly inhibited in a time- and dose-dependent manner (Fig. 1C). Concomitantly, 14-3-3σ was up-regulated and CDK1 was down-regulated upon YYJD treatment (Fig. 1D).

By extending the CCK8 cell viability assay and cell cycle analysis to additional two lung cancer cell lines (H460 and H1975), we noted similar inhibitory effect of YYJD on their proliferation (Supplemental Fig. 1A,B).

### YYJD inhibits colony formation of lung cancer cells

**Figure 2:**
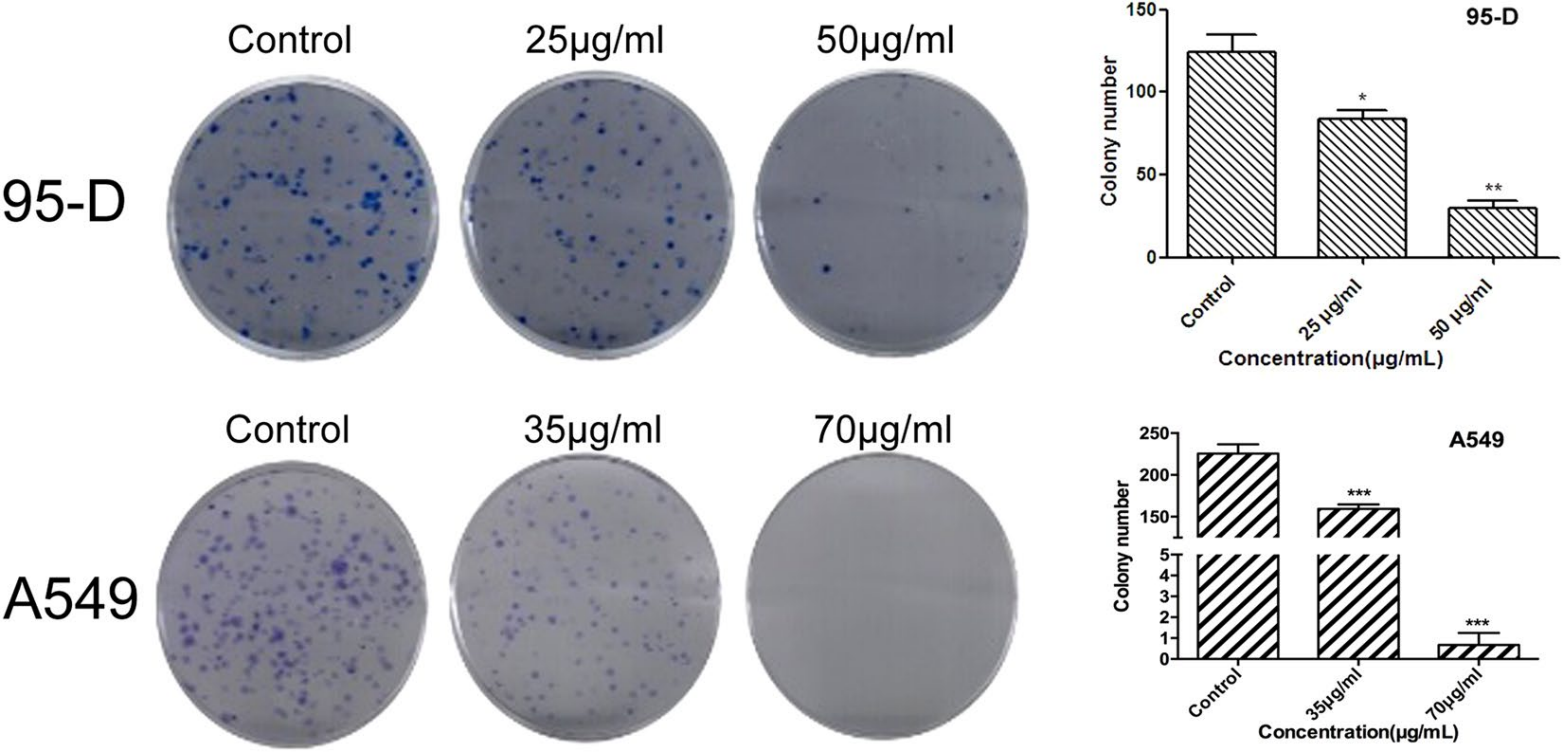
The effect of YYJD on the colony formation ability of 95-D and A549 cells. YYJD inhibits the colony formation of lung cancer cells in a concentration-dependent manner. Data are shown as mean ± SD from at least three independent experiments. *P < 0.05 and **P < 0.01 compared with control group (culture medium only).

To examine whether YYJD has an inhibitory effect on the colony formation of lung cancer cells, 95-D and A549 were treated with YYJD at different concentrations. Comparing with control, YYJD markedly suppressed the colony formation of two lung cancer cell lines in a concentration-dependent manner (Fig. 2). The colony formation of H460 and H1975 cells was also markedly reduced following the treatment of YYJD (Supplemental Fig. 1C).

### YYJD triggers premature A549 and 95-D cell senescence

**Figure 3:**
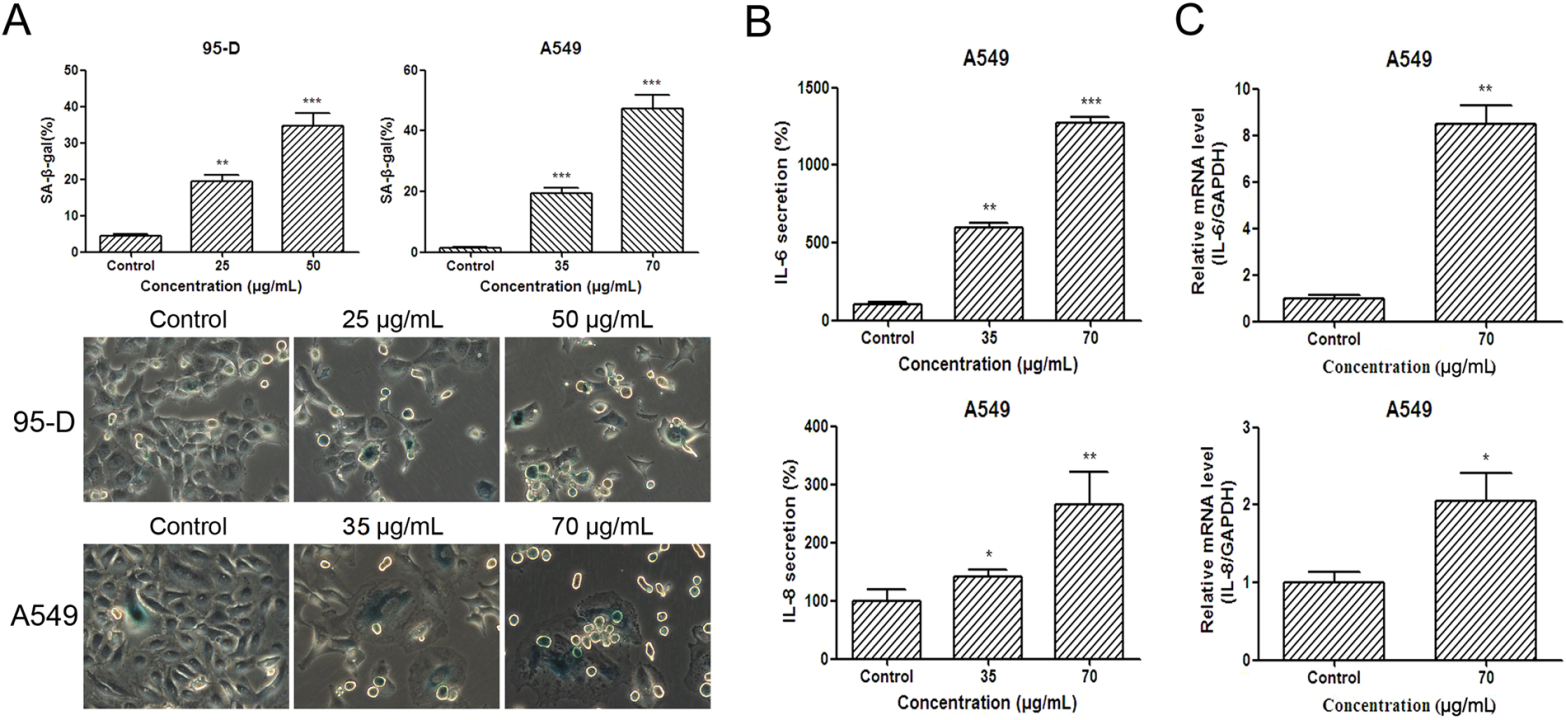
YYJD induces premature senescence and the increase of IL-6 and IL-8 in lung cancer cells. (**A**) Senescence-associated β-galactosidase (SA-β-gal) activity. The cells after YYJD-treated show more positive cells (green color) compared with the control by SA-β-gal staining. Quantitative analysis of cellular senescence from SA-β-gal staining shows that the percentage of positive cells is markedly higher in YYJD-treated cells. (**B**) ELISA analysis of IL-6 and IL-8 in A549 cells. (**C**) qT-PCR analysis of IL-6 and IL-8 mRNA in A549 cells. Data are obtained from three independent experiments and shown as mean ± SD. *P < 0.05, **P < 0.01 and ***P < 0.001 compared with control group (culture medium only).

Senescence is associated with permanent cell cycle arrest^14^. To examine the effect of YYJD on cellular senescence, 95-D and A549 cells were stained by SA-β-gal, a marker of senescent cells. After intervening with YYJD, the SA-β-gal positive cells were significantly increased in a concentration-dependent manner compared with control (Fig. 3A).

Senescent cells secrete multifold factors including the inflammatory cytokines such as IL-6 and IL-8, which can change tissue microenvironment^15–17^. These cytokines and chemokines accelerate the cell senescence by the mechanism of autocrine and paracrine^17, 18^. We found that IL-6 and IL-8 proteins and their mRNA levels were significantly increased in A549 cells after treatment with YYJD (Fig. 3B,C). Due to the low baseline in 95-D, we could not determine if IL-6 and IL-8 proteins and their mRNA levels were also increased in 95-D cells.

### YYJD increases reactive oxygen species (ROS) level and DNA damage in lung cancer cells

**Figure 4:**
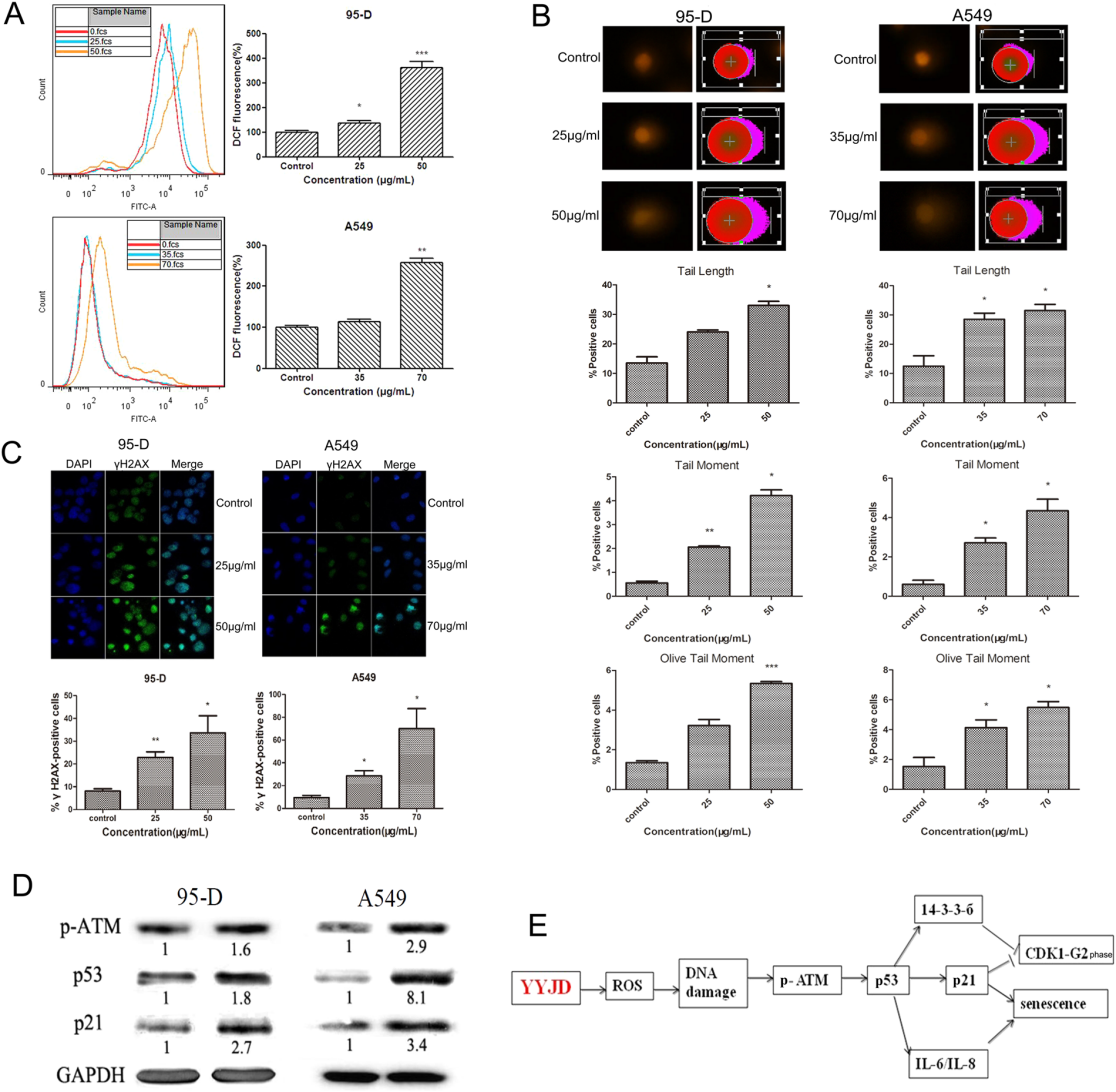
YYJD treatment leads to DNA damage and increases the generation of ROS. (**A**) The measurement of ROS. ROS levels were detected by dichlorofluorescein (DCF) measurement and flow cytometric analysis after YYJD treatment for 24 h. The ROS levels are showed as fold change compared with control cells in 95-D and A549 cells. (**B**) DNA damages in 95-D and A549 cells after exposed to YYJD for 24 h. Comet images (200×) in alkaline gel electrophoresis with 95-D and A549 cells were taken under fluorescence microscopy by using PI staining. Percentage of comet-positive cells is presented as the mean ± SD. (**C**) DNA damages were determined by γH2AX immunofluorescent microscopy. After being exposed to YYJD for 24 h, γH2AX foci immunofluorescent images (400×) in anti-γH2AX monoclonal antibody staining and DAPI nuclei staining assays were taken under immunofluorescent microscopy in 95-D and A549 cells. The γH2AX foci are appeared as bright fluorescent spots in the merge images. The percentage of γH2AX foci positive cells in YYJD-treated 95-D and A549 cells is presented as mean ± SD. (**D**) Western blot analysis of p-ATM, p53 and p21 levels. Cells were treated with YYJD for 24 h and cellular extracts subjected to Western blot. (**E**) Representative schematic diagram summarizing the signaling pathway for cancer cell cycle arrest and senescence induced by YYJD. The data are shown as mean ± SD of three independent experiments. *P < 0.05, **P < 0.01 and ***P < 0.001 compared with control group (culture medium only).

ROS plays a key role in resulting DNA damage and inducing cell senescence. We examined whether YYJD induces DNA damage and cell senescence through enhancing ROS level in lung cancer cells. To this end, we performed DCFH-DA staining and flow cytometric assays and found that the level of ROS was significantly increased upon YYJD treatment in 95-D and A549 cells (Fig. 4A). It has been reported that ROS induces DNA damage^19, 20^. To examine whether YYJD has an effect on the DNA damage, we conducted comet assay. Our results indicated that tail length, tail moment and olive tail moment were increased after the treatment of YYJD in a concentration-dependent manner (Fig. 4B). To verify the effect of YYJD on DNA damage, we examined several DNA damage-related proteins. Both γH2AX and p-ATM were increased significantly following treatment with YYJD. The expressions of p21 and p53 were notably increased after treatment by YYJD in both 95-D and A549 cells (Fig. 4C,D). These results suggest that YYJD can induce DNA damage in lung cancer cells.

### YYJD suppresses tumor growth *in vivo*

**Figure 5:**
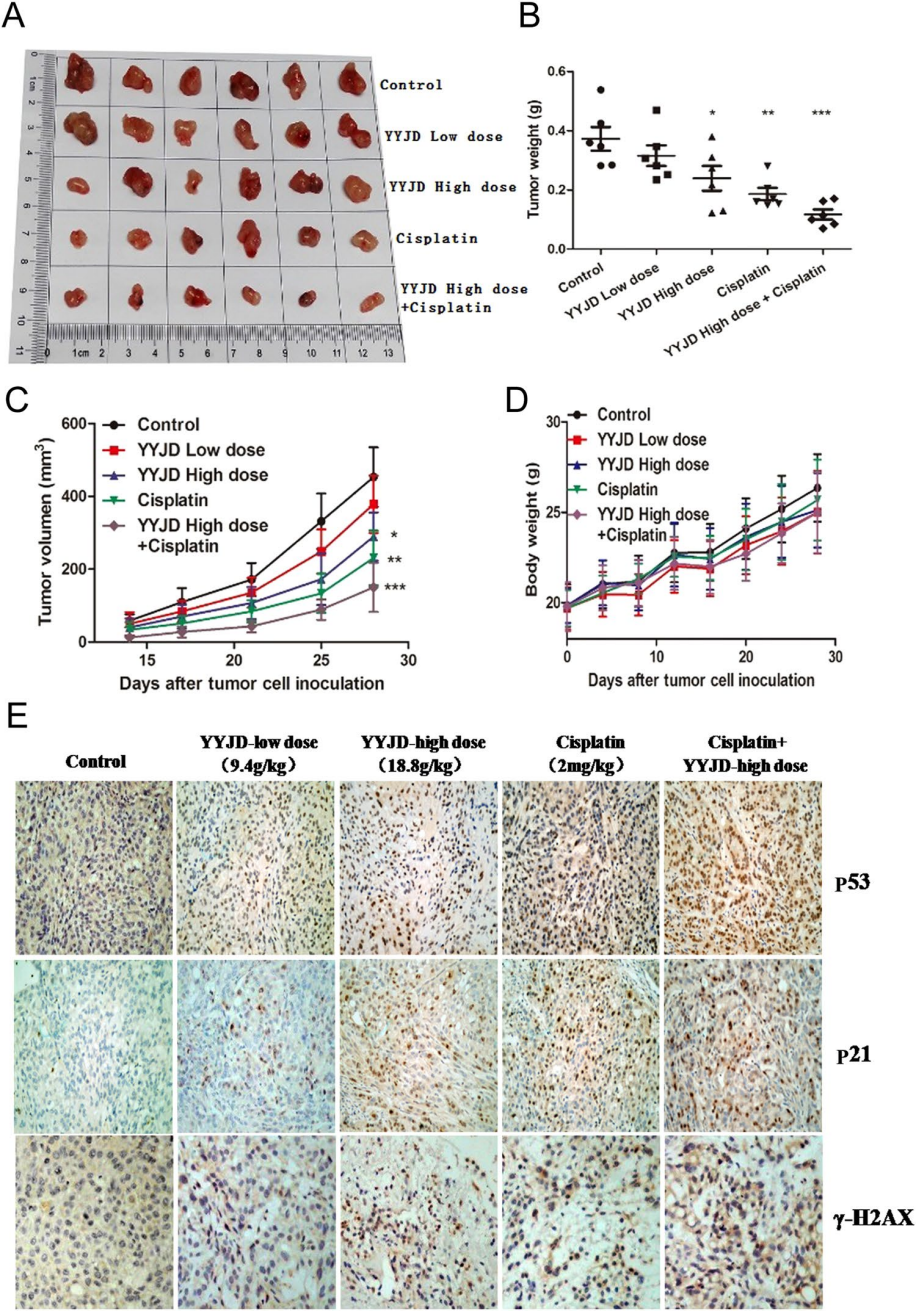
Tumor inhibitory effect of YYJD *in vivo*. 1 × 10^6^ A549 cells/mouse were subcutaneously inoculated into nude mice. The mice were randomized into 5 groups (6 nude mice/group), and treated with YYJD (9.4 g/kg, 18.8 g/kg), cisplatin (2 mg/kg, once every 4 days and in a total of four times), YYJD (18.8 g/kg) combined with cisplatin (2 mg/kg, once every 4 days and in a total of four times) and 0.9% normal saline once a day for 28 days as previously mentioned. (**A**) The tumors were excised from animals after treatment. (**B**) The comparison of tumor weights of five groups. *P < 0.05, **P < 0.01 and ***P < 0.001 indicated significant differences compared with normal saline group. (**C**) The tumor volumes were measured once every 4 days. (**D**) The weight of mice was measured once every 4 days. (**E**) The expression of p53, p21 and γH2AX in tumor xenograft tissues were detected by immunohistochemistry (400×).

Lastly, we examined the anti-tumor activity of YYJD *in vivo*. A549 cells were used to generate *s*.*c*. xenograft tumors in nude mice. YYJD administered orally suppressed the growth of lung cancer cell xenograft compared with control mice given saline. When in combination with cisplatin, the anti-tumor effect of high concentration of YYJD was stronger than that of high concentration of YYJD or cisplatin only. No significant change in animal body weight following YYJD treatment (Fig. 5A–D). An increase in p53, p21 and γH2AX was noted in xenograft (Fig. 5E). In addition, YYJD caused no change in body weight, liver and kidney function in normal mice without xenograft (Supplemental Fig. 2)

## Discussion

Chinese Herb Medicine is one of the resources worthy to be explored for new treatment of cancer^21^. However, systematic investigation is necessary to ensure the practice of Chinese Herb Medicine to be evidence-based^22–24^. In this study, we assessed the anti-tumor effect of YYJD and found that it could inhibit lung cancer cell growth, cause cell cycle G2/M arrest and senescence by inducing a signaling cascade including changes in ROS/DNA damage/p-ATM/p53/p21/14-3-3σ pathway (Fig. 4E). However, as the increase in IL-6 and IL-8 was noted only in A549 cells not 95-D cells, it is yet to determine if YYJD triggers senescence in 95-D cells through IL-6 and IL-8 independent mechanisms, or due to very low baseline of IL-6 and IL-8 in 95-D cells making it impossible to detect any increase. In addition, we found that YYJD in combination with cisplatin markedly inhibited the growth of subcutaneous human A549 xenograft tumors.

Cell senescence can play an important role in inhibiting oncogenesis^25–27^. Cellular senescence can be induced by cellular stress, such as oxidative stress or DNA damage agents^14^. Oxidative stress can result in DNA damage and the accumulation of DNA damage was deemed to be the main trigger of the cell senescence^28^. In this study, we showed that YYJD results in DNA damage, which was confirmed by the increase of ROS, γH2AX and the phosphorylation level of ATM in lung cancer cells. DNA damage has been related to the increase of cytokines and chemokines secretion, which is also associated with senescence^15, 29^. We observed that the treatment of YYJD induced markers of cell senescence, including positive SA-β-gal staining, and increase of IL-6 and IL-8. We consider that YYJD-induced cell senescence might be associated with the activation of ROS-dependent DNA damage.

The increase of p21 is important in senescence and accounts for regulation of the transcriptional activation of diversified genes which are indispensable for the induction of the senescent phenotype^30^. In various cancer types, p53 is a salient tumor suppressor and is activated when cells undergo cellular stress, such as DNA damage^31^. p53 activation is vital for the induction of senescence^32, 33^. The 14-3-3σ protein is very important in cancer biology through intervening in cell cycle checkpoints. Induction of 14-3-3σ protein expression in cancer cells can lead to inhibition of cell growth and causes cell cycle arrest at G2/M phase^34^. It has been reported that the increase of CDK1 kinase activity can also contribute to the G2/M phase cell cycle arrest^35^. Our study supports that the treatment of YYJD induces cell cycle arrest through up-regulation of 14-3-3σ, down-regulation of CDK1 and senescence through elevated expression of p53 and p21 in lung cancer cells.

In summary, our study has demonstrated that YYJD induces cell cycle arrest and senescence in lung cancer cells, ROS-dependent DNA damage and the upregulation of p21, p53, IL-6 and IL-8. Our study provides an insight of YYJD anti-tumor activities and suggests that YYJD is a potential anti-tumor drug for the treatment of lung cancer.

## Methods

### Preparation of YYJD

The raw herbs were obtained from Huayu pharmacy company in Shanghai. The five herbs of YYJD, Astragalus, Radix Ophiopogonis, Paris polyphylla, Glossy Privet Fruit, Fiveleaf Gynostemma were mixed and smashed according to the weight ratio of 3:1:2:1:1. Then 5 time volumes of 70% alcohol and 30% pure water were added and extracted with ultra sonication for 3 times (60 min each time). The supernatant was collected, and the alcohol was removed through rotary evaporation, and then dried into powder by freeze drying. For *in vitro* experiments, the YYJD powder was dissolved in culture medium. The culture medium without YYJD was used as control. HPLC was used to examine the chemical composition of YYJD extract (Supplemental Fig. 3)

### Cells, chemicals and reagents

Human lung cancer cell lines 95-D and A549, human normal lung cells BEAS-2B and 16HBE were purchased from Cell bank of Chinese academy of sciences of Shanghai. A549 was cultured in F12K medium, 95-D, BEAS-2B and 16HBE cells were cultured in RPMI1640 medium, with 10% fetal bovine serum (FBS, Gibco, USA), 100U/ml penicillin and 100 mg/ml streptomycin in a humidified atmosphere with 5% CO_2_ at 37 °C. The cells with 80% confluence were treated by YYJD of different concentrations.

The reference compounds for the chromatographic analysis were purchased from Chengdu Pusi Biotechnology Company. Antibodies against p-ATM, p53, p21, CDK1, 14-3-3σ were obtained from Santa Cruz Biotechnology (Santa Cruz, CA, USA). Cisplatin was purchased from Longhua Hospital of Shanghai, China.

### Animals

Female BALB/c nude mice (4 weeks old, weighing 18–22 g) were maintained under specific pathogen-free conditions with constant temperature (23 ± 2 °C) and controlled light (12 h light:12 h dark). All experiments were performed in accordance with relevant guidelines and regulations. The study was approved in Sino-British SIPPR/BK LAB animal Ltd (Animal authorization reference number: SCXK2013-0016).

### Analysis of number of viable cells

The cells were seeded in 96-well plates at a density of 5000 cells/well overnight, then treated with YYJD at different concentrations for 24 h, 48 h and 72 h, respectively. At each time point, Cell Counting Kit 8 (CCK8) agent (Dojindo, Japan) was added to each well and incubated for 1 h at 37 °C. The numbers of viable cell were calculated by detecting the optical density (OD) at 450 nm using the spectrometer. The IC50 values of YYJD were calculated by Graphpad software. Cell viability (%) = OD_treated_/OD_control_ × 100.

### Cell colony formation assay

The cells were seeded at 400 cells/well in six-well plates and treated with different concentrations of YYJD for 10 days. Then fixed with 4% paraformaldehyde for 1 h and stained with Giemsa dye for 30 min at room temperature. The numbers of colony were scanned and counted with scanner.

### Cell cycle analysis

The cell cycle was detected by using flow cytometry (FCM) with propidium iodide (PI) staining. Cells were seeded in six-well plates and treated with YYJD at different concentrations for 24 h followed by fixation in ice-cold 80% ethanol at 4 °C overnight. Then washed with cold PBS for one time, and added with 100 μl RnaseA for 30 min at 37 °C, the cells were suspended in PI Staining Buffer at 4 °C for 30 min, finally analyzed on a flow cytometer.

### IL-6 and IL-8 ELISA assay and SA-β-gal assay

Cells were seeded in six-well plates and intervened with YYJD at different concentrations for 24 h. The cell supernatant was collected and conducted according to the instructions of the human interleukin-6 (IL-6) or interleukin-8 (IL-8) ELISA Kit (BioLegend, USA), and finally detected at the microplate reader. Cell senescence was measured according to the protocol of the SA-β-gal assay kit. Following the protocol, after treatment with YYJD for 24 h, cells were fixed with 2% paraformaldehyde for 15 min at room temperature, finally added with SA-β-gal solution and incubated at 37 °C without CO_2_ overnight. Senescence cells were dyed blue at the microscope. The senescence index was assessed on the basis of the ratio of SA-β-gal-positive cells/total cells × 100%.

### Western blot

The cells were treated with YYJD for 24 h and lysed by RIPA buffer (Beyotime, China) for 30 min on ice, then centrifuged at 12000 rpm for 15 min at 4 °C and the supernatant was collected. The concentration of protein was measured by the BCA method (Beyotime, China). Equal amount of protein (30 μg) from each sample was separated by SDS-PAGE and transferred onto PVDF membranes. The membranes were blocked with 5% skimmed milk for 2 h at room temperature and incubated with different primary antibodies (1:1000 dilutions) overnight at 4 °C. After washing with TBST (10 mM Tris-HCL pH 7.4, 150 mM Nacl, 0.05% Tween 20) for three times, the membranes were incubated with the appropriate horseradish peroxidase-conjugated secondary antibodies (1:2000 dilutions). The protein bands were detected by using an enhanced chemiluminescence system. The protein quantitative analysis was conducted by using the Image J software.

### ROS assay

The level of intracellular ROS was detected by using dichlorofluorescein diacetate (DCFH-DA). The cells were collected after intervening and suspended in DCFH-DA, incubated for 20 min at 37 °C. The excess DCFH-DA was wiped out by washing three times with serum-free medium and 100 μl PBS was added. The fluorescence intensity was detected at 488 nm excitation wavelength and 525 nm emission wavelength with flow cytometry.

### Neutral single-cell gel electrophoresis (SCGE)

The DNA damage was detected by comet assay which was conducted as described by Singh and Olive. Three agarose layers were needed for per gel bonding. The bottom layer consisted of 1% normal melting point agarose which was dropped on the preheating (45 °C–70 °C) frosted glass slides, then cover glass was used to cover it quickly and left for 15 minutes at 4 °C. The cover glass was removed after agarose solidification, 50 μl cell suspension and 325 μl 0.65% low melt agarose were mixed at 37 °C, half of the mixture was transferred onto the normal melting point agarose of the bottom layer as the second layer and then covered with the cover glass and left at 4 °C for 15 minutes. Ensure that the second layer as thin as possible and the cells as far as possible on a plane. Then the cover glass was removed and 60 μl 0.65% low melt agarose was dropped onto the second layer at 4 °C for 15 minutes as the third layer. The Gel containing the samples was lysed in 2.5 M Nacl, 10 mM Tris, 100 mM EDTA, 1%Triton X-100 and 10% dimethyl sulfoxide at 4 °C for 2 h. The cover glass was placed in electrophoresis solution of 0.3 M NaOH, 1 mM EDTA for 20 minutes to denature (25 minutes at 4 °C and 25 V, 300 mA). Then the slides were washed and left to dry in the air, stained with silver stain. Finally, the slides for each sample were observed with a fluorescent microscope.

### Immunofluorescence analysis

Cells were seeded on coverslips, after treatment with YYJD for 24 h, fixed in 4% paraformaldehyde for 10 min, permeabilized with 1% Triton-X100 for 15 min, and blocked with 5% BSA for 2 h at 4 °C. After that, cells were incubated with primary antibody against γH2AX at 4 °C overnight, then incubated with FITC-conjugated or Rhodamine Red-conjugated secondary antibody at room temperature for 2 h, stained with DAPI. Finally detected with a confocal microscope^36^.

### Tumor growth assays

1 × 10^6^ A549 cells in 200 μl saline were injected subcutaneously into the right flank of BALB/c nude mice. The next day after injection, animals were randomly divided into 5 groups (6 mice/group) including saline, low concentration of YYJD (9.4 g/kg), high concentration of YYJD (18.8 g/kg), cisplatin (2 mg/kg) and high concentration of YYJD plus cisplatin. Chinese herbs and saline were administered via gavage. Cisplatin was injected through i.p. with 200 μl. The mice of cisplatin groups were treated once every 4 days and in a total of four times. The saline and YYJD groups were administered every day. Tumor size was measured twice every week and the volume was calculated as follows: Volume = 0.5 length × width^2^. On the 28th day after the treatment, all mice were anesthetized and the tumors were peeled off and fixed in formalin for immunohistochemistry.

### Immunohistochemical analysis

The tumor tissues were fixed in 4% paraformaldehyde solution, embedded in paraffin permeabilized with 1% Triton-X100 for 15 min, washed with PBS for three times. The tissues were first incubated with primary antibodies against p53, p21 and γH2AX, and then incubated with a secondary antibody, according to the manufacturer’s instructions.

### Statistical analysis

The data are presented as the mean ± standard deviation (SD), every experiment was performed at least 3 times. The differences between the groups were performed by using one-way ANOVA. Values of P < 0.05 were considered to indicate a statistically significant difference.

## Electronic supplementary material

Supplementary Information
