# Supplementary material for: Herbal formula YYJD inhibits tumor growth by inducing cell cycle arrest and senescence in lung cancer
Source: Sci Rep. 2017 Jul 10;7:4984. doi: 10.1038/s41598-017-05146-x (PMC5504005; doi:10.1038/s41598-017-05146-x)
Supplement: Supplementary file 1 — Supplementary Information [file 41598_2017_5146_MOESM1_ESM.pdf]

Herbal formula YYJD inhibits tumor growth by inducing cell cycle arrest and senescence in lung cancer

Tingting Zheng[1,2], Zujun Que[1], Lijing Jiao[1], Yani Kang[3], Yabin Gong[2], Jialin Yao[2], Chao Ma[1], Ling Bi[2], Qihan Dong[4], Xiaodong Zhao[3*], Ling Xu[1,2*]

1 Tumor Institute of Traditional Chinese Medicine, Longhua Hospital, Shanghai University of Traditional Chinese Medicine, 725 South Wanping Rd, shanghai 200032, China

2 Department of Oncology, Yueyang Hospital of Integrated Traditional Chinese and Western Medicine, Shanghai University of Traditional Chinese Medicine, 110 Ganhe Rd, shanghai 200437, China

3 School of Biomedical Engineering and Bio-ID Center, Shanghai Jiao Tong University, 800 Dongchuan Rd, Shanghai 200240, China

4 Endocrinology Section, Central Clinical School and Charles Perking Center, The University of Sydney. School of Science and Health, The Western Sydney University, Australia

*These corresponding authors contributed equally to this work. Correspondence and requests for materials should be addressed to L.X. (xulq67@aliyun.com)

Methods

Animal study. Kunming mice were randomly divided into 2 groups (6 mice/group), YYJD (18.8g/kg) or saline were administered through gavage daily over 14 days. On the 14th day following gavage, the blood was taken to measure the glutamic-pyruvic transaminase (ALT), glutamic oxalacetic transaminase (AST), creatinine (Cre) and urea nitrogen (BUN). Body weight was measured on day 1, 4, 7 and 10.

HPLC. Chromatographic analysis was conducted on an Alliance HPLC system. The chromatographic separation was performed with Unitary C18 column (4.6×250 mm, 5μm) using water+0.01% TFA (A) and acetonitrile+0.01% TFA (B) with the following gradient profile:0-5 min, 5-5%B; 5-30 min, 5-95%B; 30-40 min, 95-95%B. The mobile phase consisted of 0.1% formic acid (A) and 100% acetonitrile (B) and at a flow rate of 1mL/min within 40 min. The volume of injection was 10μl. The UV detection was carried out at 203 nm and 254 nm. The ELSD conditions were set as following: drift tube temperature 70℃, gain value 100, nebulizer temperature 30℃ and gas pressure 30 psi[1]. The concentrations of main chemical ingredients were determined with an external standard curve[2-4].

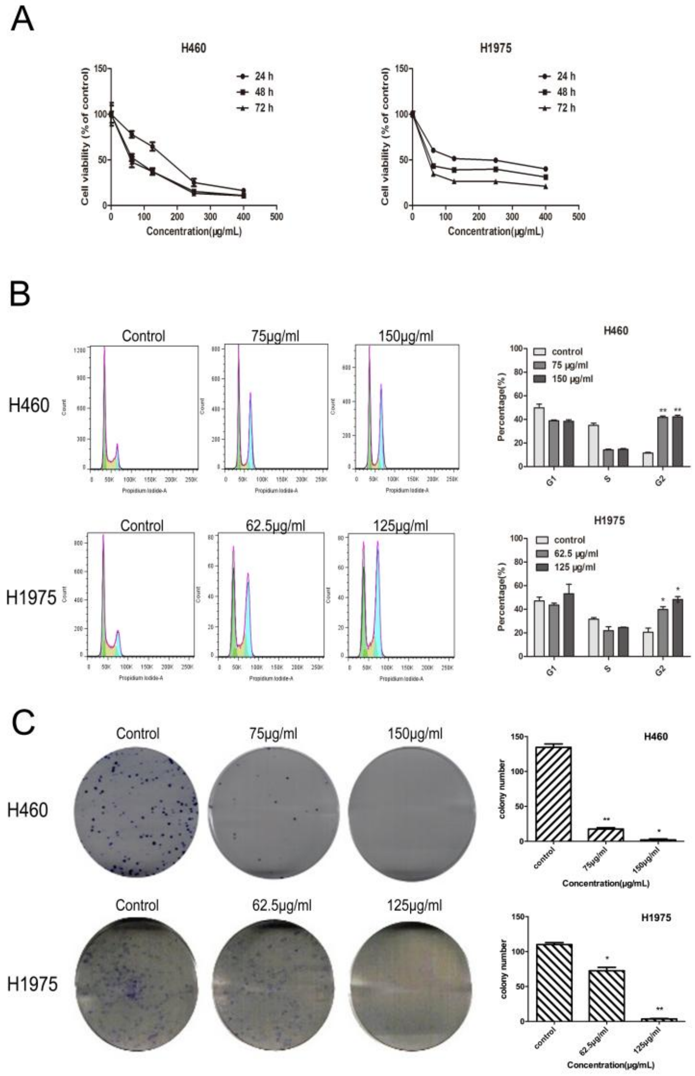

Supplemental Figure 1. YYJD inhibited the proliferation and the colony formation of lung cancer cell lines (H460 and H1975). (A) The effect of YYJD on cell viability by CCK8 assay. (B) YYJD induced cell cycle arrest at G2/M phase by flow cytometry. (C) YYJD inhibited the colony formation. The results are expressed with the mean ± SD for at least three independent experiments. **P*<0.05, ***P*<0.01 and ****P*<0.001 compared with control group (culture medium only).

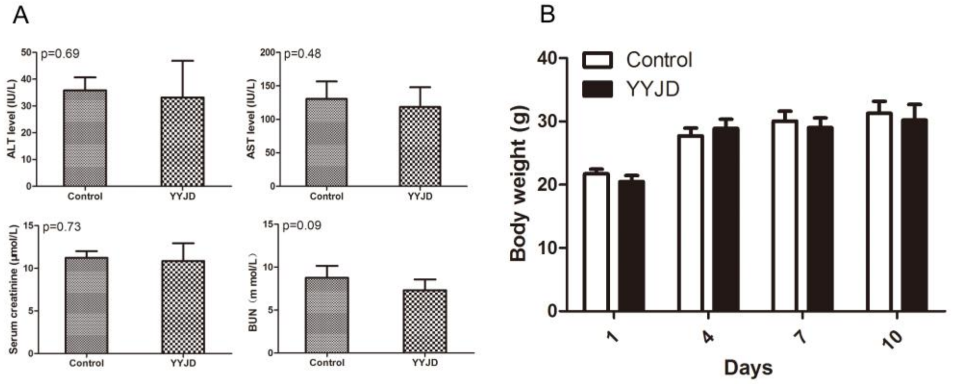

Supplemental Figure 2. The effects of YYJD on hepatic, renal function and body weight in mice. (A) ALT, AST, Cre and BUN levels in plasma. (B) The body weight of mice. Control: animals administered with saline; YYJD: animals administered with YYJD (18.8g/kg). All animals were treated for 14 days. All data are expressed as mean±SD (n=6). *P*>0.05, in comparison with the control group.

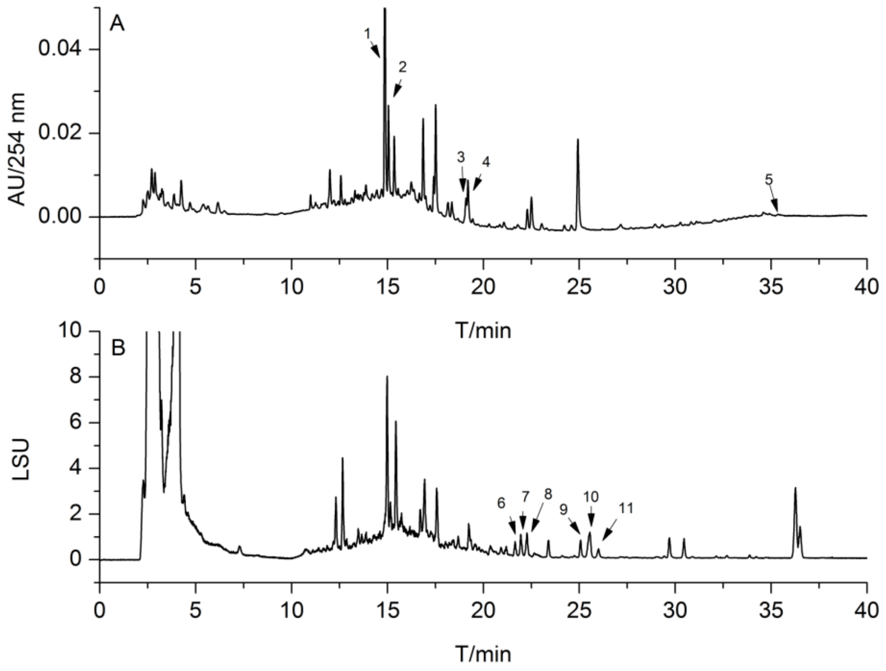

Supplemental Figure 3. HPLC fingerprint of YYJD in ethanol extract. (A) UV detection. (B) ELSD detection. 1.Rutin, 2.Calycosin-7-glucoside, 3.Calycosin, 4.Quercetin, 5.Ursolic Acid, 6.ParisSaponin VII, 7.*β*-D-glucopyranoside,(3*β*,25*R*)-17-hydroxyspirost-5-en-3-yl*O*-6-deoxy-*α*-*L*-mannopyranosyl-(1→2)-*O*-[*α*-*D*-glucopyranosyl-(1→3)], 8.Paris H, 9.Ophiopogonin D, 10.Gracillin and 11.Polyphyllin I.

References

1. Teng, Z. Y. et al. Ancient Chinese Formula Qiong-Yu-Gao Protects Against Cisplatin-Induced Nephrotoxicity Without Reducing Anti-tumor Activity. Sci Rep. **5**, 15592 (2015).

2. Hamsin, D. E., Hamid, R. A., Yazan, L. S., Che, N. M. & Ting, Y. L. The hexane fraction of Ardisia crispa Thunb. A. DC. roots inhibits inflammation-induced angiogenesis. BMC Complement Altern Med. **13**, 1-9 (2013).
3. Choi, R. C. et al. A chinese herbal decoction, danggui buxue tang, stimulates proliferation, differentiation and gene expression of cultured osteosarcoma cells: genomic approach to reveal specific gene activation. Evid Based Complement Alternat Med. **2011**, 1956-63 (2011).
4. Liao, C. R. et al. Analgesic and Anti-Inflammatory Activities of Methanol Extract of Ficus pumila L. in Mice. Evid Based Complement Alternat Med. **2012**, 340141(2012).
